# The Rôle of Modern Dietetics in the Causation of Disease

**Published:** 1906-06

**Authors:** 


**The Rôle of Modern Dietetics in the Causation of Disease** By J. Sim Wallace, M.D., B.Sc., L.D.S. Pp. vii., 87. London: Baillière, Tindall & Cox. 1905.

This is an interesting volume of essays, which affords a wide field for thought and some useful suggestions. Disease, the writer suggests, largely depends upon the individual being placed in conditions different from those which obtained during the evolution of his species. Food at the present day is to a great extent deprived of the cellulose and other inert matter for dealing with which the alimentary canal has been adapted. It is almost pure nutriment, and likely, therefore, to be unsuited to the organism. Mastication and salivary digestion under these circumstances tend to cease, and predigested foods are devised to replace them. Sugar is taken in a concentrated and irritating form; fats which are found finely divided in natural substances are eaten in a mass, or mixed with starch, so that they are digested with difficulty. Excessive quantities of nutriment at frequent intervals are taken into the stomach with the aid of condiments in consequence of this craze for concentrated foods. There is a tendency to feed healthy children and adults on the predigested foods devised for diseased states, and to diet men on the genito-urinary products of the lower animals, such as eggs and milk. A child, after the first dentition, is a gnawing animal, and naturally would consume much inert matter; but we give him concentrated starch, sugar, and meat, which results in dyspepsia, constipation, and pharyngeal congestion. Hence too maldevelopment of the palate, nose, and jaws, adenoids, loss of the perspiratory functions of the nasal passages and dental caries. In short, we feed a child to-day on pap and sodden food, though nature gives him a relatively larger dental apparatus than she does to adults. This food stimulates neither the digestive glands nor the muscles of the intestines, and yet we are startled at the presence of physical deterioration.

A difficulty in applying the writer's theories is the selection of some reasonably hard, bulky food for children before they have learned the habit of bolting their food unmasticated, a habit which the present soft concentrated food renders universal.

